# The role of hesperetin on osteogenesis of human mesenchymal stem cells and its function in bone regeneration

**DOI:** 10.18632/oncotarget.15473

**Published:** 2017-02-18

**Authors:** Deting Xue, Erman Chen, Wei Zhang, Xiang Gao, Shengdong Wang, Qiang Zheng, Zhijun Pan, Hang Li, Ling Liu

**Affiliations:** ^1^ Department of Orthopaedics, 2nd Affiliated Hospital, School of Medicine, Zhejiang University, Hangzhou 310009, P.R. China; ^2^ Department of Nephrology, Hangzhou Hospital of Traditional Chinese Medicine, Hangzhou 310007, P.R. China

**Keywords:** hesperetin, mesenchymal stem cells, bone regeneration, osteogenesis, gelatin sponge scaffold

## Abstract

Hesperetin has been suggested to be involved in bone strength. We aimed to investigate the effects of hesperetin on the osteogenic differentiation of human mesenchymal stem cells and its related mechanisms. We showed that hesperetin promoted osteogenic differentiation of human mesenchymal stem cells *in vitro*. It potentially exerts its effects via the ERK and Smad signaling pathways. Using a rat osteotomy model, we showed that human mesenchymal stem cells combined with a hesperetin/gelatin sponge scaffold resulted in accelerated fracture healing *in vivo*. Due to the low cost of hesperetin, it could be used as a growth factor for bone tissue engineering or surgical fracture treatment.

## INTRODUCTION

Fracture nonunion remains a challenging clinical problem for orthopedic surgeons. According a previous report, the delayed union and nonunion of open fractures occurs in approximately 4.4% of all fractures [1]. In the presence of a soft tissue injury, the rate of delayed union ranges from 13 to 16% [2, 3].

Several treatment options exist for this clinical problem; however, they are complex and costly, and multiple procedures are often required [4]. Autologous bone grafting remains the gold standard for the treatment of fracture nonunion, but it is associated with donor site morbidity. Commercially available recombinant human bone morphogenetic protein-2 (BMP-2) has been reported to accelerate fracture healing [5]. However, the high cost of BMP-2 limits its application. Thus, finding a new method to accelerate bone healing with therapeutic efficiency and low cost is warranted.

Hesperetin (3′,5,7-trihydroxy-4-methoxyflavanone) is a metabolite of hesperidin (hesperetin-7-O-rutinoside), which belongs to the flavanone subgroup and is found mainly in citrus fruits. Hesperetin has several biological activities including antioxidant, anti-inflammatory, analgesic, and lipid-lowering effects [6–8]. In recent years, various authors have suggested that hesperetin is involved in bone strength [9, 10]. Kim et al. [11] further showed that hesperetin alleviated the inhibitory effects of high glucose on osteoblastic differentiation of periodontal ligament stem cells. However, no study has investigated the effects of hesperetin on osteogenic differentiation of mesenchymal stem cells (MSCs), which are the main cells involved in fracture healing after injury.

MSCs have the potential to differentiate into a variety of cell types including osteoblasts, chondrocytes, and adipocytes [12]. After a fracture occurs, the local microenvironment stimulates the recruitment, proliferation, and differentiation of MSCs for initial fracture healing [13], and MSCs play a key role during fracture healing. Several studies have attempted to enhance the osteogenic potency of MSCs with the addition of osteogenic growth factors such as BMP-2 [14]. However, the high cost of commercially available growth factors limits their applications. Thus, finding a low-cost osteogenic growth factor is demanded.

The scaffold is also an important factor in bone regeneration. A suitable scaffold provides a three-dimensional structure that mimics the extracellular matrix, allowing cell attachment, migration, and proliferation [15]. Several scaffolds have been used in bone tissue engineering [16–18]. So far most researches focused on natural biomaterials such as collagen, chitosan, gelatin, chondroitin and hyaluronic acid [16, 17, 19]. As a commercially available natural material, gelatin sponges offer excellent biocompatibility, a porous structure and biodegradability. So it has good feasibility in clinical application.

In the present study, we studied the effects of hesperetin on the proliferation and osteogenic differentiation of MSCs as well as its related mechanisms *in vitro*. Through an *in vivo* study, we found that hesperetin-loaded gelatin sponge scaffolds promoted fracture healing. Due to the low cost of hesperetin, it could be used as an osteogenic factor for clinical application.

## RESULTS

### Hesperetin in low concentrations promotes the proliferation of hMSCs

**Figure 1:**
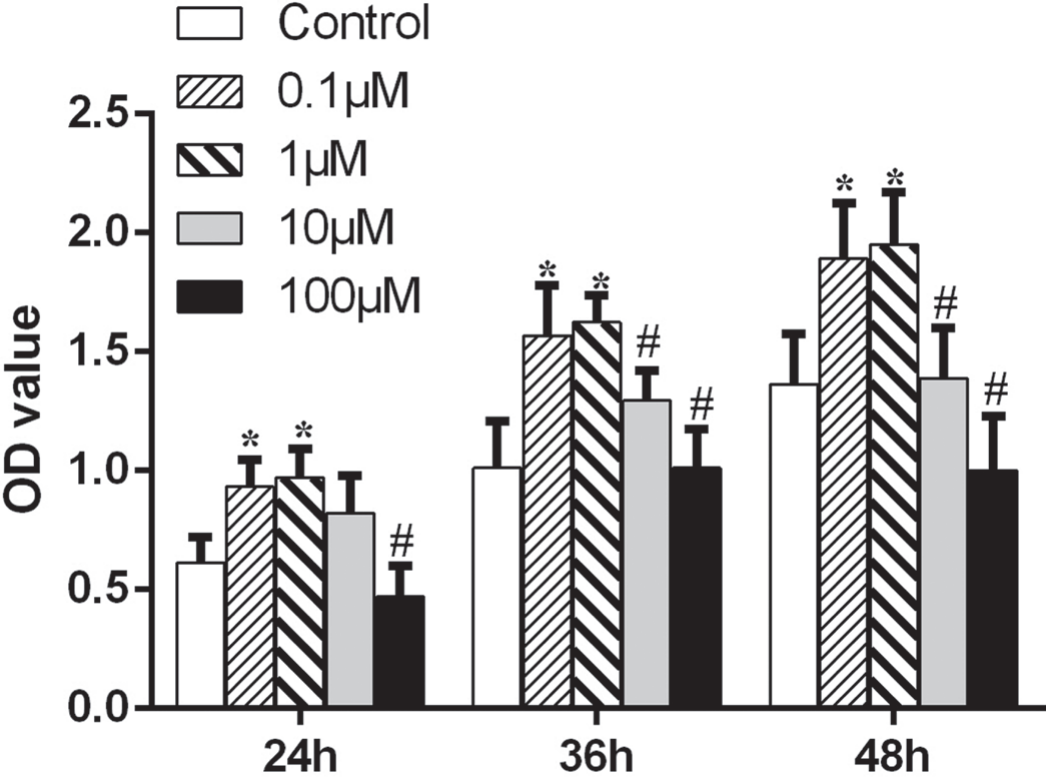
The effect of different concentrations of hesperetin on hMSC expansion *p < 0.05 vs. the control group. ^#^*p* < 0.05 vs. the lower concentrations (0.1 and 1 μM).

To study the effects of hesperetin on the proliferation of hMSCs, a Cell Counting Kit-8 (CCK-8) test was performed. Hesperetin promoted hMSCs proliferation at lower concentrations (0.1 and 1 μM) (*p* < 0.05); this effect was concentration-dependent. However, when MSCs were treated with high concentrations of hesperetin (10 or 100 μM), proliferation was inhibited (Figure 1).

### Hesperetin promotes hMSC migration

**Figure 2:**
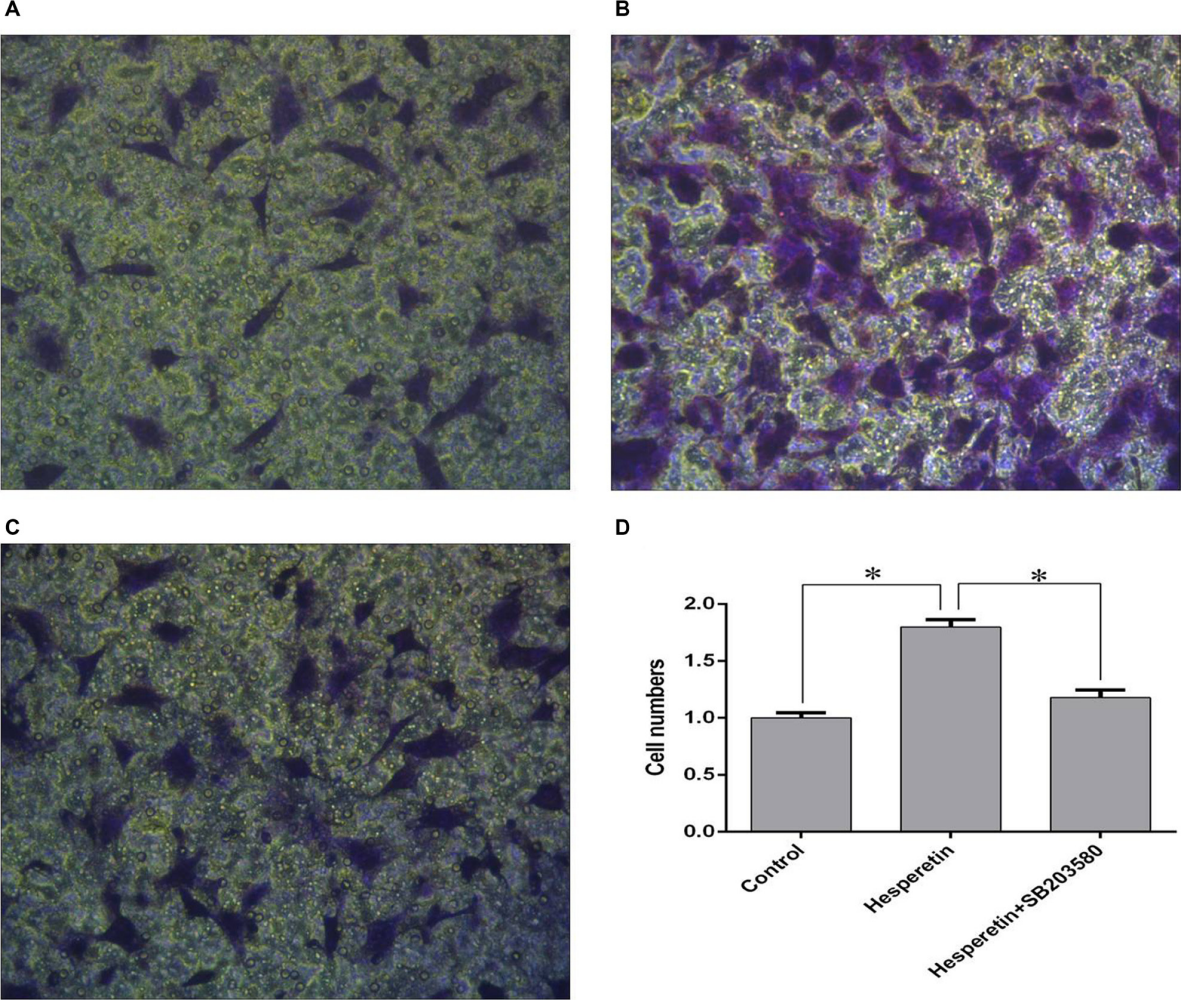
The effects of hesperetin on hMSC migration (**A**) Control group without hesperetin in the lower chamber. (**B**) Hesperetin group with hesperetin (1 μM) in the lower chamber. (**C**) Hesperetin (1 μM) co-treatment with the p38 inhibitor SB203580 in the lower chamber. Cells were pretreated with SB203580. (**D**) The number of cells located on the underside of the membrane. *p < 0.05.

A transwell study was performed to study the effects of hesperetin on the migration of hMSCs. According to the proliferation assay, a concentration of 1 μM was used for further experiments. The results showed that hesperetin promoted hMSCs migration (1.8 ± 0.06 relative to control), and co-treatment with the p38 inhibitor SB203580 inhibited the effects of hesperetin on hMSC migration (Figure 2).

### The effect of hesperetin on the osteogenetic differentiation of hMSCs

**Figure 3:**
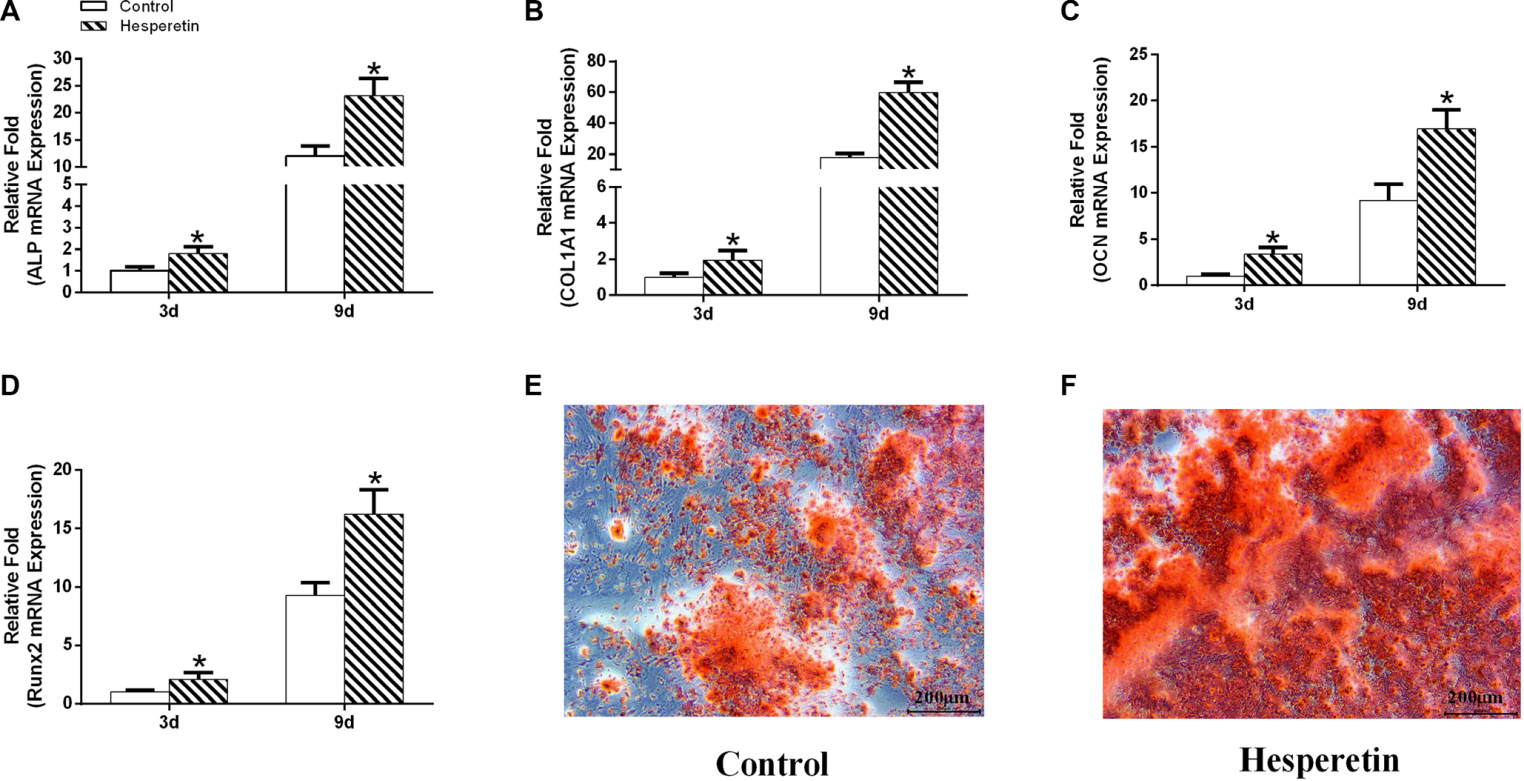
The gene expression levels of ALP, COL1A1, OCN, and Runx2 in the control and hesperetin (1 μM) groups on days 3 and 9 (**A**–**D**). The hMSCs were stained with alizarin red S on day 14 (**E**–**F**). *p < 0.05 vs. the control group.

The effect of hesperetin on the osteogenetic differentiation of hMSCs was assessed using real time PCR (RT-PCR). hMSCs were treated with 1 μM hesperetin during osteogenic induction and compared to non-treated controls. There was a significantly higher expression of ALP, runt-related transcription factor 2 (RUNX2), OCN, and COL1A1 in the hesperetin group than in the control group on days 3 and 9 (Figure 3A–3D).

Mineralization was accelerated in cells cultured for 14 days in the presence of 1 μM hesperetin in osteoinductive medium (OIM), and was significantly greater in hesperetin-treated hMSCs than in the control group (Figure 3E–3F).

### Hesperetin increased ERK1/2 phosphorylation in hMSCs

**Figure 4:**
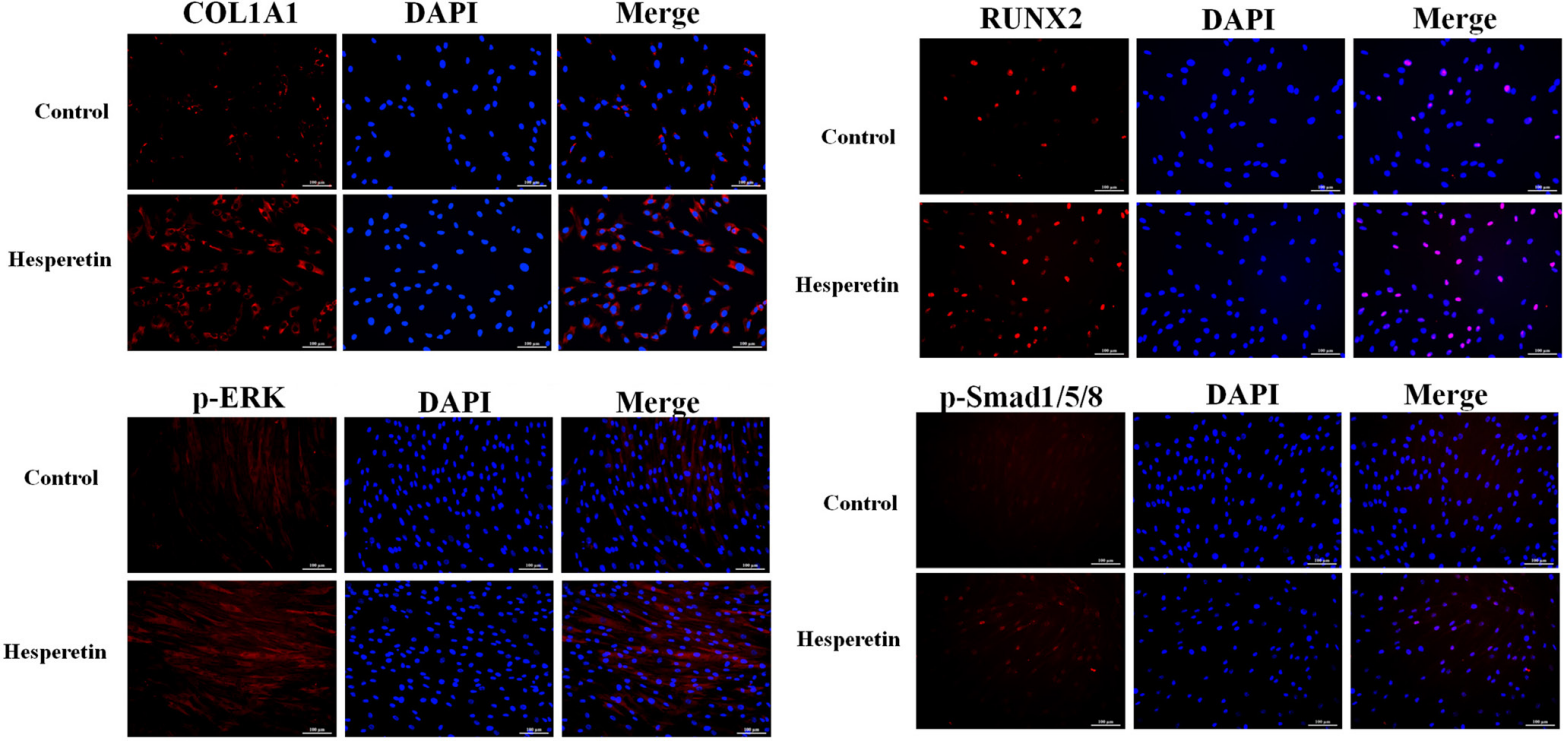
Immunofluorescence of type I collagen, runt-related transcription factor 2 (RUNX2), phosphorylated ERK (p-ERK), and phosphorylated Smad1/5/8 (p-Smad1/5/8) in hMSCs after 24-h culture in OIM with or without hesperetin (1 μM).

The ERK signaling pathway plays an important role in the regulation of hMSC osteogenesis. Thus, we investigated the potential effect of hesperetin on the ERK signaling pathway using immunofluorescence. There was an increase in RUNX2 and COL1A1 protein expression in hesperetin-treated cells 24 h after osteogenetic induction (Figure 4). After 24 h of culture in OIM, the levels of phosphorylated ERK (p-ERK) were significantly higher in the hesperetin-treated hMSC group than in the control group (Figure 4).

**Figure 5:**
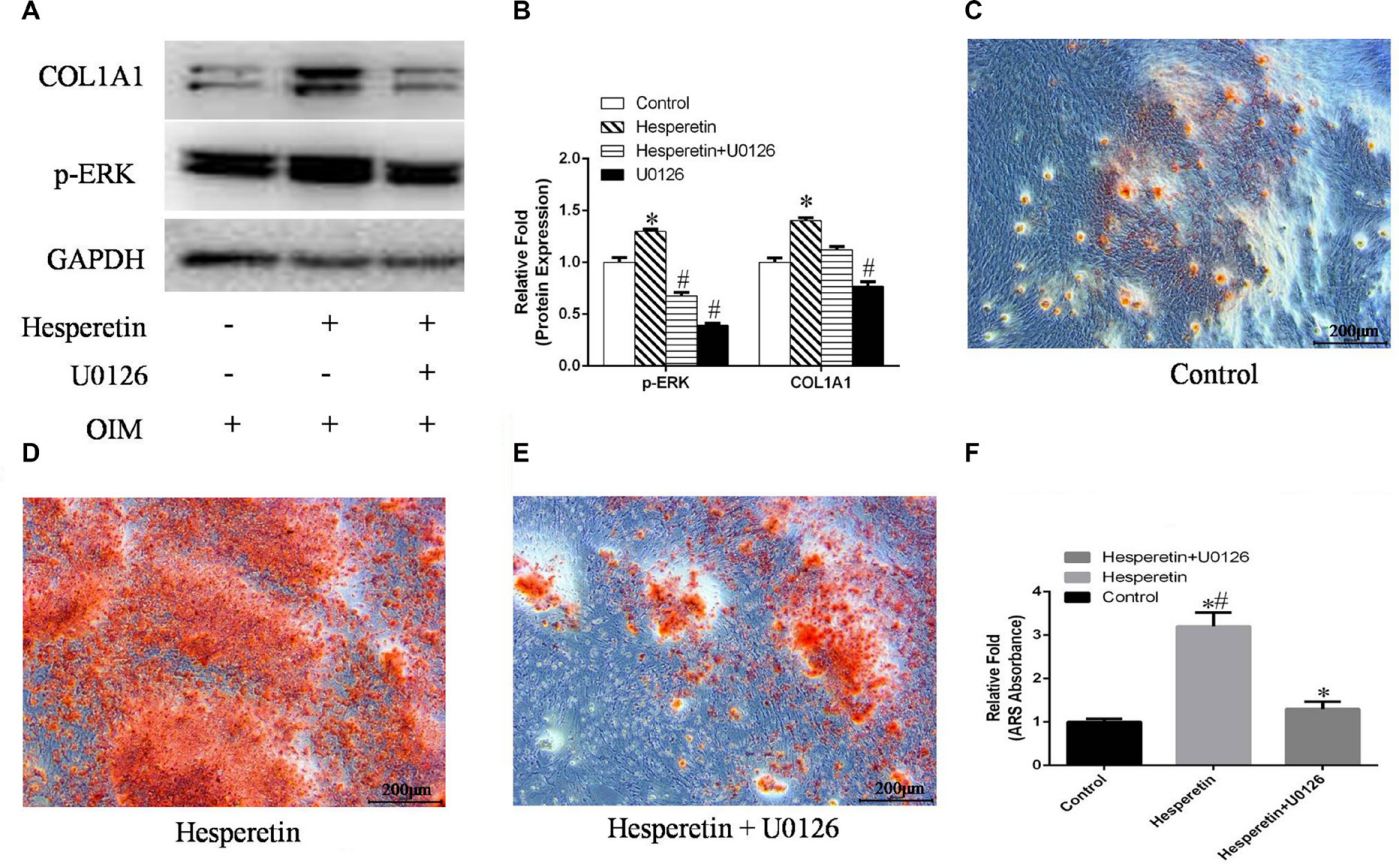
(**A**) hMSCs were cultured in OIM, OIM + hesperetin, OIM + hesperetin + U0126 or OIM + U0126, after which the expressions of p-ERK and COL1A1 were detected using Western blotting. (**B**) Protein expression levels were normalized to GAPDH. *, ^#^*p* < 0.05 vs. the control OIM group. (**C**–**E**) hMSCs were cultured in OIM, OIM + hesperetin, or OIM + hesperetin + U0126 and stained with alizarin red S (ARS) on day 9. (**F**) Mineralization was quantified with extraction of ARS-stained cells using 10% cetylpyridinium chloride (CPC). *p < 0.05 vs. OIM, ^#^*p* < 0.05 vs. OIM + hesperetin + U0126.

To further confirm the role of ERK signaling during hesperetin-induced osteogenesis, hMSCs were cultured in OIM, OIM with hesperetin, OIM with hesperetin and U0126 (an ERK inhibitor), or OIM with U0126, and Western blotting was performed. Hesperetin increased p-ERK expression, and treatment with U0126 inhibited the increase in COL1A1 protein levels induced by hesperetin (Figure 5A, 5B). Moreover, cells treated with U0126 had decreased matrix mineralization deposits at day 9 of osteogenic differentiation (Figure 5C–5F).

### Hesperetin increased phosphorylation of Smad1/5/8 in hMSCs

We next investigated the role of Smad1/5/8 in the hesperetin signaling pathway during osteogenesis, as is it an important regulator of hMSC osteogenesis. After 24-h culture in OIM, the levels of phosphorylated Smad1/5/8 (p-Smad1/5/8) were significantly higher in the hesperetin-treated hMSC group than in the control group (Figure 4).

**Figure 6:**
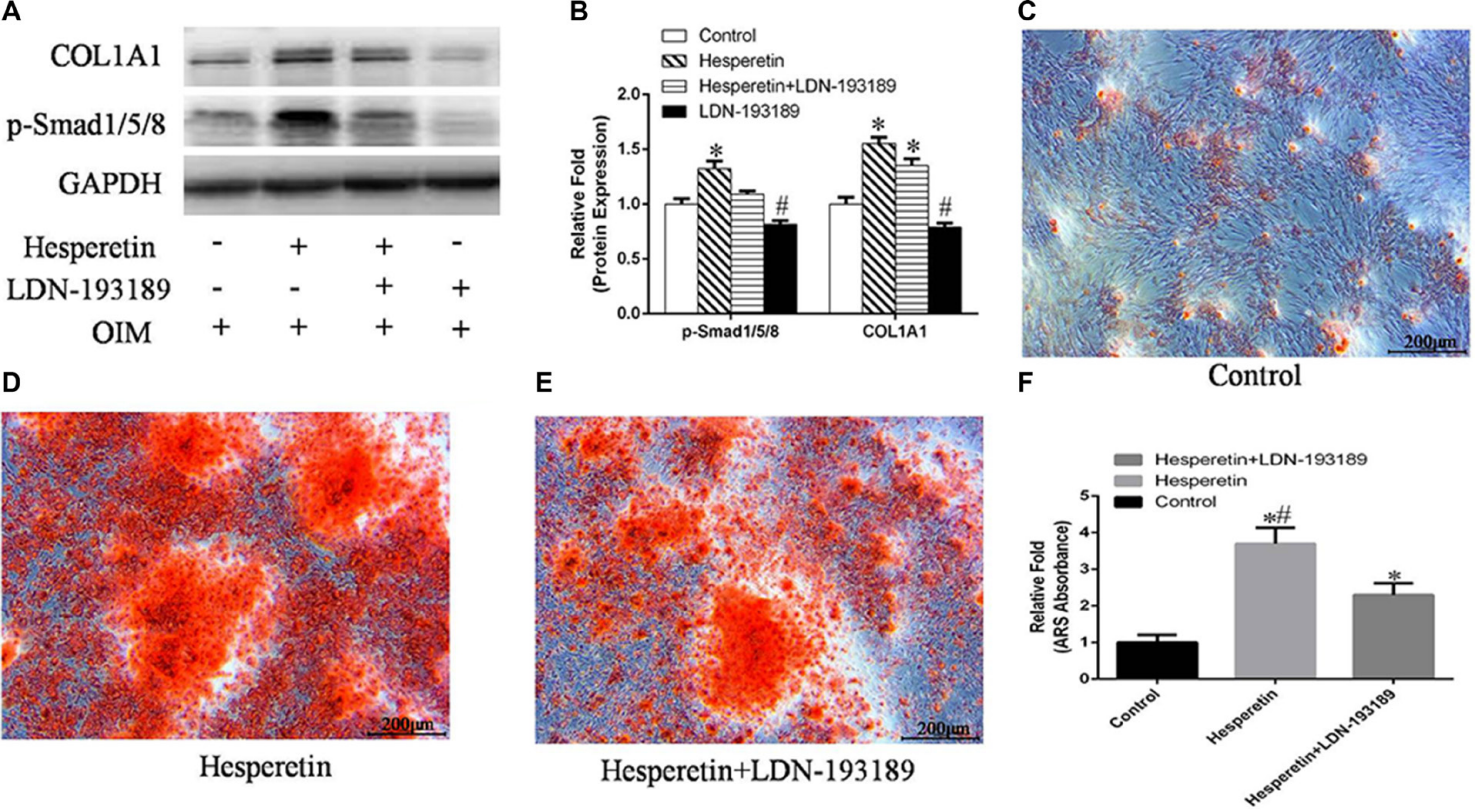
(**A**) hMSCs were cultured in OIM, OIM + hesperetin, OIM + hesperetin + LDN-193189 or OIM + LDN-193189, after which the expression of phosphorylated Smad1/5/8 (p-Smad1/5/8) and COL1A1 were detected by Western blotting. (**B**) Protein expression levels were normalized to GAPDH. *, ^#^*p* < 0.05 vs. the control OIM group. (**C**–**E**) hMSCs were cultured in OIM, OIM + hesperetin, or OIM + hesperetin + LDN-193189 and stained with alizarin red S (ARS) on day 9. (f) Mineralization was quantified by the extraction of ARS-stained cells using 10% cetylpyridinium chloride (CPC). *p < 0.05 vs. OIM, ^#^*p* < 0.05 vs. OIM + hesperetin + LDN-193189.

To further confirm the role of Smad1/5/8 signaling during hesperetin-induced osteogenesis, hMSCs were cultured in OIM, OIM with hesperetin, OIM with hesperetin and LDN-193189 (a Smad1/5/8 inhibitor) or OIM with LDN-193189 and Western blotting was performed. Hesperetin increased p-Smad1/5/8 expression, and LDN-193189 co-treatment with hesperetin inhibited the increase of COL1A1 protein levels (Figure 6A, 6B). Moreover, cells treated with LDN-193189 had decreased matrix mineralization deposits at day 9 of osteogenic differentiation (Figure 6C–6F).

### The effect of hesperetin/gelatin sponge scaffold on hMSC expansion

**Figure 7:**
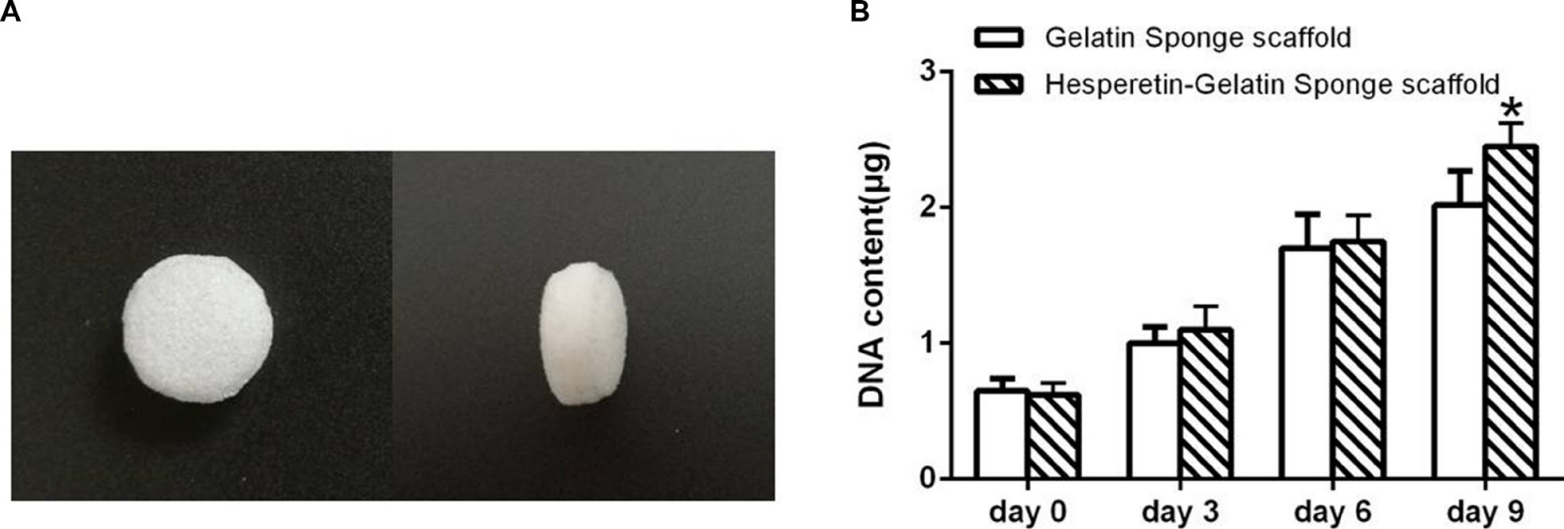
(**A**) Disc-shaped gelatin sponge scaffolds. (**B**) The DNA content of MSCs in the gelatin sponge and hesperetin/gelatin sponge scaffolds after 0, 3, 6, and 9 days of culture. Each scaffold was seeded with 100 μL of cells to achieve a density of 1 × 10^6^ cells/scaffold. *p < 0.05 between the hesperetin/gelatin and gelatin sponge scaffolds at the same time point.

The proliferation of MSCs in scaffolds was assessed using a DNA assay with Hoechst33258 dye. By day 9, the DNA content of the cells entrapped in the gelatin sponge and in the hesperetin/gelatin sponge scaffolds had increased 3.11 and 3.95 fold, respectively, compared with day 0. On day 9, the DNA content of the cells in the hesperetin/gelatin sponge scaffold (2.45 ± 0.17 μg) was significantly higher than that of cells in the gelatin sponge scaffolds (2.02 ± 0.25 μg) (*p* = 0.029), indicating that hesperetin can improve gelatin sponge scaffold biocompatibility to support the proliferation of hMSCs *in vitro* (Figure 7).

### hMSCs combined with hesperetin/gelatin sponge scaffolds accelerated rat tibial fracture healing

To study the effect of hesperetin/gelatin sponge scaffolds on fracture healing *in vivo*, a tibial osteotomy model was created in rats and subsequently repaired with hesperetin/gelatin-hMSC composites, hesperetin/gelatin scaffolds, gelatin scaffolds alone, or left untreated as a control. All rats used in this study survived the experimental period until they were euthanized. The wounds healed well without signs of infection and did not limit the range of motion.

**Figure 8:**
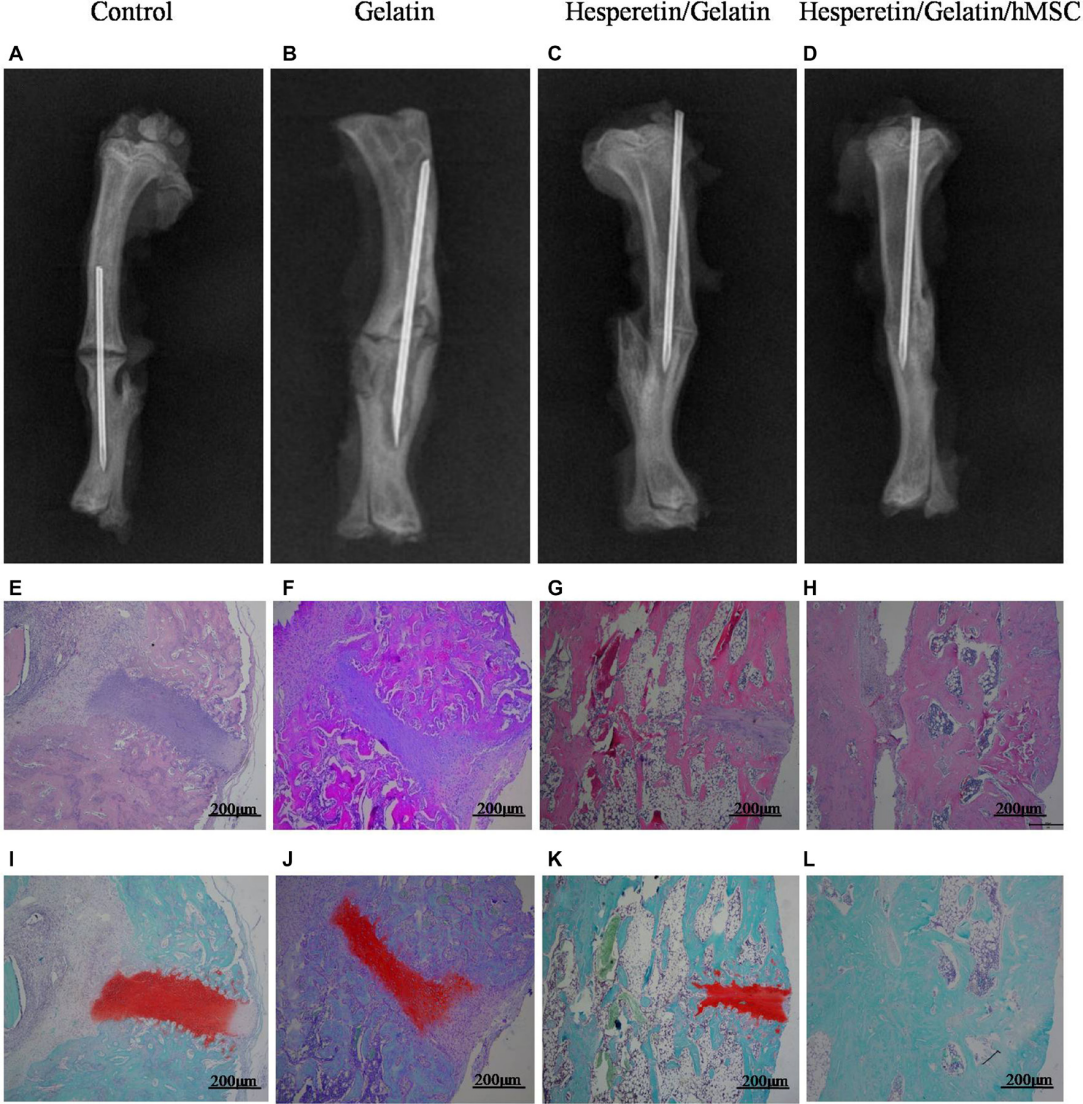
(**A**–**D**) Radiographic images of osteotomized rat tibias 8 weeks post-operatively. (**E**–**L**) Hematoxylin and eosin and Safranin-O staining of osteotomized rat tibias 8 weeks post-operatively. Staining was evaluated at ×50 magnification.

At 8 weeks postoperatively, the fracture line was apparent, and only minimal bone growth in the control and gelatin sponge scaffolds groups were seen (Figure 8A, 8B). Bridging of the osseous defects was seen in the hesperetin/gelatin-hMSC and hesperetin/gelatin groups (Figure 8C, 8D); in the former group, complete fracture union was achieved without a cortical gap.

Fracture healing was confirmed histologically using hematoxylin and eosin-stained (HE) and Safranin-O-stained tibias of the four groups at 8 weeks postoperatively. The control and gelatin sponge scaffold groups remained at the beginning stage of endochondral ossification (Figure 8E–8J). However, the hesperetin/gelatin-hMSC and hesperetin /gelatin groups were at the end stage of endochondral ossification (Figure 8G, 8K, 8H, 8L). Fracture healing was almost finished in the hesperetin/gelatin-hMSC group (Figure 8H, 8L).

**Figure 9:**
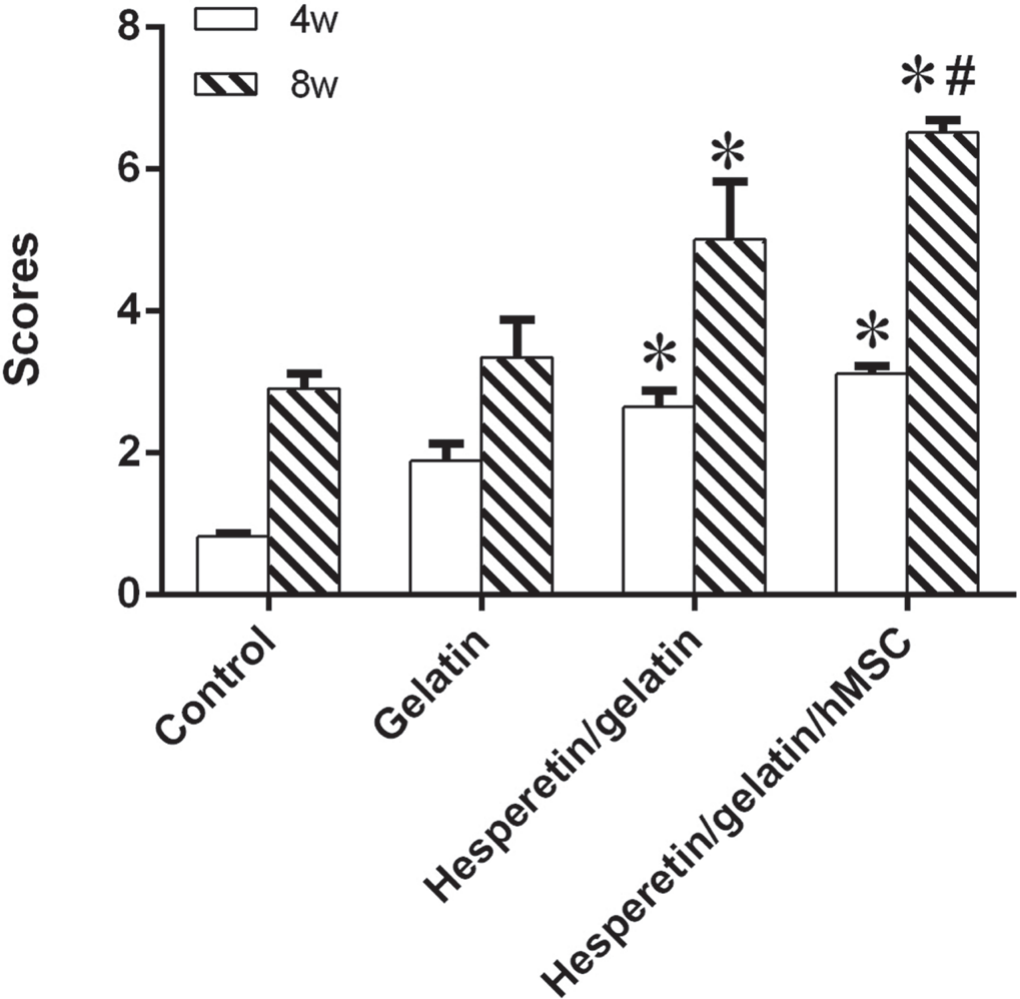
Radiographic scores of fracture sites, *P < 0.05 vs the control and gelatin groups, ^#^*P* < 0.05 vs. the hesperetin/gelatin group.

In order to quantitative analysis, a scoring scale which was based on rebridgement of the cortices was used. Our results showed that the fracture healing in hesperetin/gelatin group (5.01 ± 0.81) and hesperetin/gelatin-hMSC group (6.52 ± 0.18) were significant higher than control (3.35 ± 0.53) and gelatin spongy groups (2.91 ± 0.21) at 8 weeks postoperatively (Figure 9).

## DISCUSSION

Compromised bone healing remains a challenging clinical problem for orthopedic surgeons. In this study, we showed that hesperetin promoted hMSC proliferation and osteogenic differentiation through the ERK and Smads signaling pathways. To the best of our knowledge, this is the first study to demonstrate that local delivery of hesperetin improves fracture healing in a rat tibial osteotomy model.

Several studies have demonstrated that flavonoids stimulate bone metabolism to improve bone mineral density [20, 21]. Hesperetin, a member of the flavanone subclass of flavonoids, is found mainly in citrus fruits. Kim et al. [11] found that hesperetin alleviated the inhibitory effect of high glucose on the osteoblastic differentiation of periodontal ligament stem cells. Trzeciakiewicz et al. [22] showed that hesperetin stimulated the differentiation of primary rat osteoblasts. However, to date, no study has been performed investigating the role of hesperetin on MSC osteogenic differentiation and fracture healing. In this study, low concentrations of hesperetin promoted hMSC proliferation and osteogenic differentiation. It is interesting to note that when MSCs were treated with high concentrations of hesperetin, proliferation was inhibited. Thus, the effects of hesperetin on MSCs may be related with the doses used; thus, we used 1 μM hesperetin for all subsequent osteogenic differentiation experiments. Hesperetin (1 μM) accelerated MSC osteogenic differentiation. To date, no agreement has been reached on an effective concentration of hesperetin for *in vitro* or *in vivo* studies. Kim [11] compared hesperetin concentrations ranging between 0.1 and 100 μM, and showed that osteogenic differentiation increased with increasing concentrations, but peaked at a concentration of 0.1 μM. Trzeciakiewicz [22] performed an *in vitro* study using hesperetin concentrations of 1 and 10 μM. They showed that 1 and 10 μM hesperetin activated the Smad signaling pathway. Thus, a concentration of hesperetin lower than 10 μM may exert effects in *in vitro* studies. For our *in vivo* study, we applied 100 μL of hesperetin at a concentration of 100 μM per scaffold to assess rat tibial fracture healing. After dilution in a 12 well-plate (2 ml per well), the final concentration was 5 μM. Our results showed that this hesperetin–gelatin scaffold construct supported hMSC proliferation and promoted fracture healing. Thus, 5 μM of hesperetin may be a suitable concentration for *in vivo* studies.

Several signaling pathways are involved in MSC osteogenic differentiation, including the ERK1/2, Wnt, phosphatidylinositide-3 kinase (PI3K)/Akt, and BMP-Smad pathways [23–26]. In our study, we demonstrated that the ERK1/2 and Smad1/5/8 signaling pathways were involved in the ability of hesperetin to induce MSCs osteogenic differentiation. The ERK pathway belongs to the mitogen-activated protein kinase (MAPK) pathways. It has been shown that inhibition of ERK1/2 abolishes the osteogenic response of adipose-derived stem cells [27]. Thus, ERK1/2 is necessary for osteogenesis. However, Lund et al. [28] found that inhibition of the ERK1/2 pathway led to an amplification of the osteogenic program in hMSCs. Whether ERK1/2 is stimulatory or inhibitory may depend on the specific cells and treatments studied. In our study, when we inhibited the ERK signaling pathway using U0126, calcium deposits were dramatic decreased, indicating that the ERK pathway plays an important role in hesperetin-induced osteogenic differentiation of hMSCs. The BMP-Smad pathway is another important pathway involved in osteogenesis. Numerous *in vitro* and *in vivo* studies have confirmed that activation of the BMP-Smad pathway using BMP promotes osteogenesis [29, 30]. In our study, hesperetin induced p-Smad1/5/8 expression. When we used LDN-193189 to inhibit p-Smad1/5/8 activation, mineralization and expression of collagen I were moderately inhibited. Thus, the Smad1/5/8 pathway was partially involved in the hesperetin-induced osteogenic differentiation of MSCs.

In the “diamond concept” of fracture healing, osteogenic cells, scaffolds, growth factors, and the mechanical environment are four important elements of bone regeneration [31]. In this study, we chose hMSCs as osteogenic cells due to their osteogenic potential. Through an *in vivo* study, we demonstrated that hMSCs promoted fracture healing. The possible underlying reasons for the improvement of fracture healing by MSC transplantation are the effects of MSCs themselves and the effects of the bioactive factors secreted by transplanted MSCs. The *in vivo* niche of transplanted MSCs is important, and the external signals of the fracture region control the final fate of MSCs [32]. Our previous study showed that hMSCs could survive in the lesion region when transplanted into rats *in vivo* [33]. This may due to the immunosuppressive character of MSCs. Moreover, transplanted MSCs not only responded to the stimuli and differentiated; they but also secreted bioactive factors that resulted in chemoattraction of host cells to the repair site [34]. Thus, both transplanted hMSCs and host MSCs may participate in the fracture healing processes. Several studies have emphasized the mechanical properties of scaffolds. However, in clinical applications, orthopedic surgeons typically choose internal fixation to repair broken bones. Therefore, we chose gelatin sponge scaffolds due to their commercial availability.

In our study, the hesperetin also acts as a chemotactic factor. Our *in vitro* results showed that hesperetin promoted hMSC migration. Several signal pathways involves in the cell migration [35, 36]. The p38 inhibitor SB203580 could inhibit the effects of hesperetin on hMSC migration. So the p38 MAPK signaling pathway is one of the mechanisms through which hesperetin promotes hMSC migration. Lew et al. [37] found that p38 involved in stem cell proliferation. In our study, the duration of transwell test was only four hours. So the effect of SB203580 on the proliferation of hMSC may not affect the results of transwell test. According to the *in vitro* results, we may speculate that when the hesperetin control-released *in vivo*, it may induce the migration of MSC or preosteoblastic cells from the peripheral tissue of fracture during the early phase of the healing. But there are no effective methods to confirm this speculation so far. In future, with the technology development, we may make this question clear.

Our study has several limitations. First, higher concentrations of hesperetin inhibited hMSCs proliferation. However, we only used a lower concentration (1 μM) of hesperetin to investigate the osteogenic differentiation of hMSCs and its related mechanisms. Therefore, further studies are warranted to determine the effects of different concentrations of hesperetin on hMSC osteogenic differentiation and its related mechanisms. Second, we showed that both the ERK and Smad1/5/8 signaling pathways were involved in hesperetin-induced osteogenic differentiation of MSCs. In our mineralization study, ERK signaling seemed more important than Smad1/5/8 signaling; however, it remains unclear whether crosstalk between the ERK and Smad1/5/8 pathways exists.

In conclusion, this study showed that hesperetin promotes hMSC osteogenic differentiation through ERK and Smad1/5/8 signaling. Hesperetin-loaded gelatin sponge scaffolds accelerated fracture healing. In clinical settings, the use of hesperetin-loaded gelatin spongy scaffolds may accelerate fracture healing during surgical treatment of fractures.

## MATERIALS AND METHODS

### Bone marrow MSC isolation

Human MSCs (hMSCs) were isolated from the whole bone marrow of two healthy donors at the Second Affiliate Hospital of Zhejiang University, which was washed out during the intramedullary nailing of a femoral fracture fixation, using the method described previously [40]. All donors gave informed consent prior to the collection of their bone marrow, and the protocol was approved by the Ethics Committee of the Second Affiliate Hospital of Zhejiang University. Investigation has been conducted in accordance with the ethical standards and according to the Declaration of Helsinki and according to national and international guidelines and has been approved by the authors’ institutional review board. After obtaining the suspension of bone marrow, the mononuclear cells fraction was isolated from the suspension by centrifugation over Ficoll-Paque (density 1.077 g/ml, Pharmacia, Sweden) at 2500 rpm for 30 minutes. The obtained cells were seeded in α-MEM medium (α-MEM; Gibco BRL, Hangzhou, China) supplemented with 10% fetal calf serum (FBS; Cyagen Biosciences, Guangzhou, China). They were incubated at 37°C in a high-humidity environment containing 5% CO_2_. The medium was replaced every 3 days, and the cells were passed at approximately 80% confluence. hMSCs in the third passage were prepared for the experiments described below.

### The effect of hesperetin on hMSC proliferation

hMSCs were cultured in 96-well plates (10^3^/well) and treated with 0.1, 1, 10 or 100 μM hesperetin in α-MEM for 1, 3, and 7 days. Cell proliferation was measured using a cell counting kit-8 assay (CCK-8; Dojindo, Kumamoto, Japan) following the manufacturer's instructions. Briefly, the medium was replaced with 100 μL of α-MEM and 10 μL of CCK-8 at each time point. After a 4-h incubation at 37°C, the absorbance at 450 nm was determined using a model 680 microplate reader (Bio-Rad, Hercules, CA, USA).

Hesperetin was dissolved in dimethyl sulfoxide (DMSO) immediately before use, and the final concentration of DMSO did not exceed 0.1% in any of the experiments. DMSO at the corresponding concentrations was used as a control.

### The effect of hesperetin on hMSC migration

Cell migration was examined using transwell chambers (pore size, 8-μm diameter; Corning Costar, Acton, MA, USA). Complete medium containing 0.1% FBS was added to the wells of a 24-well plate, and serum-starved hMSCs (2 × 10^5^) were suspended in 200 μL α-MEM containing 0.1% FBS and added to the upper chamber. Prior to the addition of hesperetin, the transwell plate with the MSCs in the upper chamber and medium containing 0.1% FBS only in the lower chamber was first incubated at 37^°^C for 1 h. After the addition of hesperetin, the plate was incubated at 37^°^C for 4 h, followed by membrane fixation with 4% paraformaldehyde and staining with 0.1% crystal violet (both from Sangon Biotech, Shanghai, China) for half an hour. The membranes were then washed, and the cells on the underside of the membranes were observed under a light microscope (Leica DMI LM; Leica Microsystems, Wetzlar, Germany). Subsequently, the number of cells was counted in 5–10 random fields for each membrane.

### The effect of hesperetin on hMSC osteogenic differentiation and Alizarin red staining

The osteogenic differentiation of hMSCs was investigated. To induce the osteogenic lineage, the cells were seeded in 12-well culture plates and cultured osteogenic induction medium (OIM): DMEM supplemented with 10% FBS, 100 nM dexamethasone, and 50 mg ascorbic acid 2-phosphate/mL. Osteogenic differentiation was verified by positive staining with 0.5% alizarin red S (ARS) (pH 4.1) after immobilization of the isolated cells in 4% paraformaldehyde for 10 min.

### Real-time quantitative polymerase chain reaction assays (RT-qPCR)

The hMSCs were seeded in 6-well plates (4 × 10^4^ cells/well) and cultured with OIM and 1 μM hesperetin. Total RNA was isolated from cells cultured for 3 and 9 days using the RNAiso reagent (TaKaRa Bio Inc., Shiga, Japan). Reverse transcription was performed using 2 μg of total RNA, the Prime ScriptRT reagent kit, and gDNA Eraser (TaKaRa). The mRNA levels of alkaline phosphatase (ALP), osteocalcin (OCN), runt-related transcription factor 2 (Runx2), and Collagen α1 type I (COL1A1) were determined using a StepOnePlus real-time PCR system (Applied Biosystems Inc., Carlsbad, CA, USA) and the SYBR Premix Ex Taq (TaKaRa) together with the following conditions: 95°C for 30 s followed by 40 cycles of 95°C for 5 s, 60°C for 30 s. 18S RNA was used as an internal control to adjust for differences between samples. The concentrations were calculated using the 2^–ΔΔCt^ method. All of the primers used in this experiment were synthesized by Sangon Biotech (Shanghai, China). They are listed in Table 1.

**Table 1:** Primer sequences for real-time quantitative polymerase chain reaction

Genes	Forward primer	Reverse primer
ALP	GACACGCTGAGCCTCGTCACT	CCTGGACCGTTTCCGTATAGG
RUNX2	CAAGTGGCCAGGTTCAACGA	TGTGAAGACCGTTATGGTCAAAGTG
COL1A1	CCTGCTGGCAAGAGTGGTGAT	CAAGTTCCGGTGTGACTCGTG
OCN	AGGACCCTCTCTCTGCTCAC	GCTCACACACCTCCCT
18S	GACTCAACACGGGAAACCTCAC	CCAGACAAATCGCTCCACCAAC

### Immunofluorescence

The cells were cultured under induction medium in a 12-well plate. Runx2, COL1A1, p-ERK and p-Smad1/5/9 were detected using fluorescence microscopy (Leica). Briefly, after 10 min of fixation at room temperature in 4% paraformaldehyde, the cells were blocked for 30 min in 0.04% Triton X-100 and 5% bovine serum albumin and then incubated overnight with anti RUNX2 (1:1,600; Cell Signaling Technology, Shanghai, China), anti-COL1A1 (1:500; Abcam, Cambridge, UK), anti-p-ERK (1:100; Cell Signaling Technology), anti-p-Smad1/5/8 (1:800; Cell Signaling Technology). They were then incubated with a fluorescence-conjugated secondary antibody (Beyotime, China) for 60 min. The cell nuclei were stained with DAPI (KeyGen Biotech, Nanjing, China) for 4 min. Cell immunofluorescence was observed using fluorescence microscopy (Leica, Germany).

### Western blotting

The cells were lysed in RIPA lysis buffer (Beyotime) and then subjected to SDS-PAGE on a 10% acrylamide gel. The protein bands were blotted onto a PVDF membrane (Millipore, Shanghai, China). After blocking in 10% non-fat milk for 1 h, the membrane was incubated overnight at 4°C with antibodies specific to GAPDH (1:1,500; Cell Signaling Technology), RUNX2 (1:1,600; Cell Signaling Technology), p-ERK(1:1,000; Cell Signaling Technology), or p-Smad1/5/8 (1:1,000; Cell Signaling Technology) and then with horseradish-peroxidase-conjugated goat anti-rabbit IgG (1:1,500; Cell Signaling Technology), as a secondary antibody, for 1 h at room temperature. The immunoreactive bands were detected using an enhanced chemiluminescent detection reagent (Millipore) and further visualized by exposing the blot to X-ray film (Bio-Rad) for 0.2–2 min. Protein expression was quantified by measuring the ratio of the absorbance of the proteins of interest to that of the internal control GAPDH.

### Cell seeding and construct culture *in vitro*

The sterilized gelatin sponge scaffolds were prepared by cutting absorbable gelatin sponge (porosity 80%, Nanjing Jinling, China) into discs (diameter: 5 mm, thickness: 2 mm). Then, hesperetin (Sigma) was dissolved in enthanol sulotion, and a concentration of 100 μM was used for preparing hesperetin-gelatin sponge scaffolds constructs. 100μL hesperetin sulotion was uniformly pipetted onto the gelatin sponge scaffolds (100 μL hesperetin/scaffold). The scaffolds were frozen at –80°C for 12 h and freeze-dried for 24 h.

The scaffolds were placed in the wells of a 12-well culture plate (Corning, USA). The density of MSCs was regulated to 1 × 10^6^ cells/mL. MSC aliquots of 100 μL were seeded onto each scaffold and allowed to attach for 3 hours at 37°C before being immersed in complete medium. The constructs were incubated *in vitro* at 37°C in 5% CO_2_. After 12 days, the scaffold MSCs were used in an *in vivo* study.

### The effect of the gelatin-hesperetin scaffolds on hMSC proliferation

The number of cells in the cell scaffolds was assessed by quantifying the amount of DNA contained within them using Hoechst 33258 dye (Sigma, St. Louis, MO, USA) in an assay performed on days 0, 3, 6 and 9. Briefly, the cell-scaffold constructs collected at the different time points were crushed and then incubated with proteinase K (Sigma) at 45°C overnight. The lysates (500 μL) were then treated with 500 μL of Hoechst 33258 solution (1 μg/mL, Sigma) and the fluorescence intensity at 450 nm was measured spectrophotometrically (SpectraMax M5; Molecular Devices, Sunnyvale, CA, USA). The DNA content in the samples was determined based on a calibration curve prepared with calf thymus DNA (Sigma) as the standard.

### Animals and surgery

All animal experiments were performed in accordance with the guidelines of the Animal Care and Use Committee of Zhejiang University. The 16 12- to 13-week-old male SD rats (280–320 g) were supplied by the Animal Center of Zhejiang Academy of Medical Sciences. They were group housed in a light and temperature-controlled environment and given adequate food and water.

Sixteen animals underwent 32 osteotomy procedures and were then randomly divided into four groups. The gelatin/hesperetin-MSCs group was treated with a gelatin sponge/hesperetin composite combined with MSCs (*n* = 8 tibias). Rats in the gelatin/hesperetin group received gelatin sponge/hesperetin composites (*n* = 8 tibias). A third group was treated with gelatin sponges alone (gelatin group, *n* = 8 tibias), and the control group was left untreated (*n* = 8 tibias). Histological evaluation was carried out 8 weeks (*n* = 8 tibias per group) postoperatively.

The rats were anesthetized with 0.3% pentobarbital sodium (Sigma) administered intraperitoneally at a dose of ∼2.3 mL/kg body weight (30 mg/kg body weight). A lateral incision (1.5 cm) was made on the proximal tibia and the muscle was divided longitudinally to expose the tibia. The periosteum was removed as much as possible from the proximal to the distal aspect of the tibia. An oscillating mini saw was used to perform a transverse osteotomy from front to back in the proximal one third of the tibia; the gap was 1 mm. The rats were then treated as described above. For the gelatin sponge treated groups, the gelatin sponge was put into the gap of osteotomy. Then we trimmed the excessive scaffold around the site of osteotomy. And then a small hole was drilled in the tibial tubercles to allow insertion of a 21-gauge needle until the distal end of the tibia, resulting in loose fixation. After surgery, the rats were allowed unprotected weight-bearing movement within their cages.

### Radiographic examination and Histological staining

The animals were euthanized at 4 and 8 weeks postoperatively by an enteroceliac injection of an overdose of pentobarbital sodium. The dissected tibias were examined grossly and photographed. Radiographs were taken with a dual-track molybdenum/rhodium^+^ Mo target mammography device (22 KV, 250 mAS; General Electric, Fairfield, CT, USA). For radiographic evaluation, the scout view X-ray image was saved. For radiographic evaluation, the scout view X-ray image was saved. In order to quantitative analysis, a scoring scale which was based on rebridgement of the cortices and acceleration of healing was used (Table 2). The evaluation was under a triple-blinded protocol.

**Table 2:** X-ray scoring scale of fracture healing

Scores	X-ray of fracture sites
0	No bridging, no callus formation
1	No bridging, initiation of a small amount callus
2	No bridging, obvious initial callus formation near fracture
3	No bridging, marked callus formation near and around fracture site
4	Rebridging of at least one of the cortices, marked callus formation near and around fracture site
5	Rebridging of at least one of the cortices, marked and complete callus formation around fracture site
6	Rebridging of both cortices, and/or some resolution of the callus
7	Clear rebridging of both cortices and resolution of the callus

The specimens were fixed in 10% neutral formalin, decalcified with 10% EDTA (pH 7.4), sectioned longitudinally through the medullary canal, and embedded in paraffin. Sagittal sections (3-μm thickness) were obtained from the center of the defect and stained with hematoxylin and eosin, and safranin-O.

### Statistical analysis

Statistical analysis was performed using SPSS software (ver. 17.0; SPSS Inc., Chicago, IL, USA). Statistical significance between samples were determined by the non-parametric tests Mann–Whitney and Wilcoxon. The data are presented as means ± SD. A *p value* ≤ 0.05 was considered to indicate statistical significance.

## References

[1] Phieffer LS, Goulet JA (2006). Delayed unions of the tibia. J Bone Joint Surg Am.

[2] Velazco A, Whitesides TE, Fleming LL (1983). Open fractures of the tibia treated with the Lottes nail. J Bone Joint Surg Am.

[3] Clancey GJ, Hansen ST (1978). Open fractures of the tibia: a review of one hundred and two cases. J Bone Joint Surg Am.

[4] Bell A, Templeman D, Weinlein JC (2016). Nonunion of the Femur and Tibia: An Update. Orthop Clin North Am.

[5] Govender S, Csimma C, Genant HK, Valentin-Opran A, Amit Y, Arbel R, Aro H, Atar D, Bishay M, Borner MG, Chiron P, Choong P, Cinats J (2002). Recombinant human bone morphogenetic protein-2 for treatment of open tibial fractures: a prospective, controlled, randomized study of four hundred and fifty patients. J Bone Joint Surg Am.

[6] Choi EJ (2008). Antioxidative effects of hesperetin against 7, 12-dimethylbenz(a)anthracene-induced oxidative stress in mice. Life Sci.

[7] Choi EJ, Ahn WS (2008). Neuroprotective effects of chronic hesperetin administration in mice. Arch Pharm Res.

[8] Hirata A, Murakami Y, Shoji M, Kadoma Y, Fujisawa S (2005). Kinetics of radical-scavenging activity of hesperetin and hesperidin and their inhibitory activity on COX-2 expression. Anticancer Res.

[9] Trzeciakiewicz A, Habauzit V, Mercier S, Barron D, Urpi-Sarda M, Manach C, Offord E, Horcajada MN (2010). Molecular mechanism of hesperetin-7-O-glucuronide, the main circulating metabolite of hesperidin, involved in osteoblast differentiation. J Agric Food Chem.

[10] Chiba H, Uehara M, Wu J, Wang X, Masuyama R, Suzuki K, Kanazawa K, Ishimi Y (2003). Hesperidin, a citrus flavonoid, inhibits bone loss and decreases serum and hepatic lipids in ovariectomized mice. J Nutr.

[11] Kim SY, Lee JY, Park YD, Kang KL, Lee JC, Heo JS (2013). Hesperetin alleviates the inhibitory effects of high glucose on the osteoblastic differentiation of periodontal ligament stem cells. PLoS One.

[12] Klimczak A, Kozlowska U (2016). Mesenchymal Stromal Cells and Tissue-Specific Progenitor Cells: Their Role in Tissue Homeostasis. Stem Cells Int.

[13] Gibon E, Lu L, Goodman SB (2016). Aging, inflammation, stem cells, and bone healing. Stem Cell Res Ther.

[14] Zhou N, Li Q, Lin X, Hu N, Liao JY, Lin LB, Zhao C, Hu ZM, Liang X, Xu W, Chen H, Huang W (2016). BMP2 induces chondrogenic differentiation, osteogenic differentiation and endochondral ossification in stem cells. Cell Tissue Res.

[15] Tatara AM, Mikos AG (2016). Tissue Engineering in Orthopaedics. J Bone Joint Surg Am.

[16] Noth U, Rackwitz L, Heymer A, Weber M, Baumann B, Steinert A, Schutze N, Jakob F, Eulert J (2007). Chondrogenic differentiation of human mesenchymal stem cells in collagen type I hydrogels. J Biomed Mater Res A.

[17] Chang CH, Liu HC, Lin CC, Chou CH, Lin FH (2003). Gelatin-chondroitin-hyaluronan tri-copolymer scaffold for cartilage tissue engineering. Biomaterials.

[18] Puvaneswary S, Raghavendran HB, Talebian S, Murali MR, S AM, Singh S, Kamarul T (2016). Incorporation of Fucoidan in beta-Tricalcium phosphate-Chitosan scaffold prompts the differentiation of human bone marrow stromal cells into osteogenic lineage. Sci Rep.

[19] Xia W, Liu W, Cui L, Liu Y, Zhong W, Liu D, Wu J, Chua K, Cao Y (2004). Tissue engineering of cartilage with the use of chitosan-gelatin complex scaffolds. J Biomed Mater Res B Appl Biomater.

[20] Shen CL, Chyu MC (2016). Tea flavonoids for bone health: from animals to humans. J Investig Med.

[21] Yamaguchi M (2006). Regulatory mechanism of food factors in bone metabolism and prevention of osteoporosis. Yakugaku Zasshi.

[22] Trzeciakiewicz A, Habauzit V, Mercier S, Lebecque P, Davicco MJ, Coxam V, Demigne C, Horcajada MN (2010). Hesperetin stimulates differentiation of primary rat osteoblasts involving the BMP signalling pathway. J Nutr Biochem.

[23] Simann M, Le Blanc S, Schneider V, Zehe V, Ludemann M, Schutze N, Jakob F, Schilling T (2017). Canonical FGFs Prevent Osteogenic Lineage Commitment and Differentiation of Human Bone Marrow Stromal Cells Via ERK1/2 Signaling. J Cell Biochem.

[24] Baker N, Sohn J, Tuan RS (2015). Promotion of human mesenchymal stem cell osteogenesis by PI3-kinase/Akt signaling, and the influence of caveolin-1/cholesterol homeostasis. Stem Cell Res Ther.

[25] Tornero-Esteban P, Peralta-Sastre A, Herranz E, Rodriguez-Rodriguez L, Mucientes A, Abasolo L, Marco F, Fernandez-Gutierrez B, Lamas JR (2015). Altered Expression of Wnt Signaling Pathway Components in Osteogenesis of Mesenchymal Stem Cells in Osteoarthritis Patients. PLoS One.

[26] Li A, Xia X, Yeh J, Kua H, Liu H, Mishina Y, Hao A, Li B (2014). PDGF-AA promotes osteogenic differentiation and migration of mesenchymal stem cell by down-regulating PDGFRalpha and derepressing BMP-Smad1/5/8 signaling. PLoS One.

[27] Liu Q, Cen L, Zhou H, Yin S, Liu G, Liu W, Cao Y, Cui L (2009). The role of the extracellular signal-related kinase signaling pathway in osteogenic differentiation of human adipose-derived stem cells and in adipogenic transition initiated by dexamethasone. Tissue Eng Part A.

[28] Lund AW, Stegemann JP, Plopper GE (2009). Inhibition of ERK promotes collagen gel compaction and fibrillogenesis to amplify the osteogenesis of human mesenchymal stem cells in three-dimensional collagen I culture. Stem Cells Dev.

[29] Yadav PS, Khan MP, Prashar P, Duggal S, Rath SK, Chattopadhyay N, Bandyopadhyay A (2016). Characterization of BMP signaling dependent osteogenesis using a BMP depletable avianized bone marrow stromal cell line (TVA-BMSC). Bone.

[30] Wang CL, Xiao F, Wang CD, Zhu JF, Shen C, Zuo B, Wang H, Li Wang XY, Feng WJ, Li ZK, Hu GL, Zhang XL (2016). Gremlin2 Suppression Increases the BMP-2-Induced Osteogenesis of Human Bone-Marrow-Derived Mesenchymal Stem Cells via the BMP-2/Smad/Runx2 Signaling Pathway. J Cell Biochem.

[31] Giannoudis PV, Einhorn TA, Marsh D (2007). Fracture healing: the diamond concept. Injury.

[32] Moore KA, Lemischka IR (2006). Stem cells and their niches. Science.

[33] Xue D, Zheng Q, Zong C, Li Q, Li H, Qian S, Zhang B, Yu L, Pan Z (2010). Osteochondral repair using porous poly(lactide-co-glycolide)/nano-hydroxyapatite hybrid scaffolds with undifferentiated mesenchymal stem cells in a rat model. J Biomed Mater Res A.

[34] Caplan AI, Dennis JE (2006). Mesenchymal stem cells as trophic mediators. J Cell Biochem.

[35] Kong X, Zhong M, Su X, Qin Q, Su H, Wan H, Liu C, Wu J, Shang H, Zhang Y, Lin N (2016). Tetramethylpyrazine Promotes Migration of Neural Precursor Cells via Activating the Phosphatidylinositol 3-Kinase Pathway. Mol Neurobiol.

[36] Zhang M, Sun L, Wang X, Chen S, Jia Q, Liu N, Chen Y, Kong Y, Zhang L, Zhang AL (2013). Activin B promotes BM-MSC-mediated cutaneous wound healing by regulating cell migration via the JNK-ERK signaling pathway. Cell Transplant.

[37] Lew WZ, Huang YC, Huang KY, Lin CT, Tsai MT, Huang HM (2016). Static Magnetic Fields Enhance Dental Pulp Stem Cell Proliferation by Activating The p38 MAPK Pathway as Its Putative Mechanism. J Tissue Eng Regen Med.

[38] Le Blanc K, Tammik C, Rosendahl K, Zetterberg E, Ringden O (2003). HLA expression and immunologic properties of differentiated and undifferentiated mesenchymal stem cells. Exp Hematol.

[39] Niemeyer P, Kornacker M, Mehlhorn A, Seckinger A, Vohrer J, Schmal H, Kasten P, Eckstein V, Sudkamp NP, Krause U (2007). Comparison of immunological properties of bone marrow stromal cells and adipose tissue-derived stem cells before and after osteogenic differentiation in vitro. Tissue Eng.

[40] Zong C, Xue D, Yuan W, Wang W, Shen D, Tong X, Shi D, Liu L, Zheng Q, Gao C, Wang J (2010). Reconstruction of rat calvarial defects with human mesenchymal stem cells and osteoblast-like cells in poly-lactic-co-glycolic acid scaffolds. Eur Cell Mater.

[41] Poncelet AJ, Vercruysse J, Saliez A, Gianello P (2007). Although pig allogeneic mesenchymal stem cells are not immunogenic in vitro, intracardiac injection elicits an immune response in vivo. Transplantation.

